# Perceptions regarding utilization of meteorological information in healthcare in Korea: a qualitative study

**DOI:** 10.1186/s40557-018-0214-3

**Published:** 2018-02-01

**Authors:** Minsu Ock, Eun Young Choi, Inbo Oh, Seok Hyeon Yun, Yoo-Keun Kim, Hyunsu Kim, Min-Woo Jo, Jiho Lee

**Affiliations:** 1 Department of Preventive Medicine, Ulsan University Hospital, University of Ulsan College of Medicine, Ulsan, Republic of Korea; 2 Department of Preventive Medicine, University of Ulsan College of Medicine, Seoul, Republic of Korea; 3 Environmental Health Center, University of Ulsan College of Medicine, Ulsan, Republic of Korea; 4 Department of Occupational and Environmental Medicine, Ulsan University Hospital, University of Ulsan College of Medicine, 877 Bangeojinsunhwan-doro, Dong-gu, Ulsan, 44033 Republic of Korea; 5 Department of Atmospheric Sciences, Pusan National University, Busan, Republic of Korea

**Keywords:** Meteorological concepts, Focus groups, Republic of Korea

## Abstract

**Background:**

Health forecasting has been used in an attempt to provide timely and tailored meteorological information to patients and healthcare providers so that they might take appropriate actions to mitigate health risks and manage healthcare-related needs. This study examined the in-depth perceptions of healthcare providers and the general public regarding the utilization of meteorological information in the healthcare system in Korea.

**Methods:**

The COREQ (Consolidated Criteria for Reporting Qualitative Research) checklist was applied to this study. We conducted three focus group discussions in accordance with semi-structured guidelines developed to deal with various aspects of the utilization of meteorological information in healthcare settings. The verbatim transcriptions and field notes were analyzed according to content analysis.

**Results:**

Six physicians, four nurses, three emergency medical technicians, and seven members of the general public participated in the focus group discussions. There were some individual discrepancies among most participants regarding the health effects of climate change. Although several physician participants felt that meteorological information utilization is not a prime concern during patient care, most of the general public participants believed that it should be used in the patient care process. The provision of meteorological information to patients undergoing care is expected to not only improve the effective management of climate-sensitive diseases, but also boost rapport between healthcare providers and patients.

**Conclusions:**

More attempts should be made to provide meteorological information to groups vulnerable to climate change, and the effects of this information should be evaluated in terms of effectiveness and inequality. The findings of this study will be helpful in countries and institutions trying to introduce health forecasting services.

**Electronic supplementary material:**

The online version of this article (10.1186/s40557-018-0214-3) contains supplementary material, which is available to authorized users.

## Background

Globally, the effects of climate change, which is defined as “a change in global or regional climate patterns” [1], are already being felt and is now recognized as one of the main challenges for mankind [2]. Climate change can affect food security [3] and may exacerbate poverty, particularly in low- and middle-income countries and regions [4]. More importantly, climate change can pose a variety of risks to human health. Several articles have discussed the potential health effects of climate change, such as an increased transmission of infectious disease, an exacerbation of respiratory diseases, and mental health problems due to stress [5–7]. Accordingly, protection of the population’s health from the various effects of climate change has become an emerging priority for the public health field in all countries around the world [2].

To address the health effects of climate change, diverse approaches are required [8–10]. First, it is necessary to investigate the health effects of climate change and monitor the occurrences of climate change-related diseases and injuries. It is also important to inform, educate, and empower not only healthcare providers, but also the general public about the health impacts of climate change. Furthermore, effective health communication concerning climate change between healthcare providers and the general public is needed. This communication should consider various levels of understanding, cultural differences, and distinct susceptibilities to the health impacts of climate change. Although mitigation of climate change is a fundamental solution, these approaches can enable people to adapt to the negative effects of climate change.

One such approach, health forecasting, has been an attempt to provide timely and tailored meteorological information, such as air temperature, precipitation amount, ultraviolet index, and relative humidity, to patients and healthcare providers to enable them to take appropriate actions mitigating health risks and managing healthcare-related needs [11, 12]. A popular example is *Healthy Outlook*, which was developed by the United Kingdom Meteorological Office to provide meteorological information to patients with chronic obstructive pulmonary disease (COPD) [13–15]. *Healthy Outlook* takes advantage of routine meteorological and communicable disease reports to rely health risks to COPD patients via an interactive telephone call. Health forecasting has also been applied to the development of an alert system for cold weather and heat waves [16] and to predict emergency department visits [17]. In the case of the Republic of Korea (hereafter Korea), most of the general public has acknowledged the need for health meteorological information [18, 19]. However, the opinions of healthcare providers on the utilization of meteorological information in the healthcare profession need to be confirmed to adapt and expand health forecasting in Korea. Additionally, a more in-depth perception of the general public about these issues, which cannot be detected using a quantitative study, needs to be explored using qualitative methods [20].

In the current qualitative study, we examined the in-depth perceptions of healthcare providers and the general public regarding the utilization of meteorological information in the healthcare system in Korea.

## Methods

We conducted three focus group discussions (FGDs) to assess the perceptions of healthcare providers and the general public regarding the utilization of meteorological information in healthcare. We have detailed the methods used in this qualitative study by applying the COREQ (Consolidated Criteria for Reporting Qualitative Research) checklist [21].

### Organization of the research team

The research team was comprised of seven members. Three of the team members have considerable experience in conducting qualitative studies. Six of the team members have participated in other studies related to climate and environmental issues.

### Study participants and reflexivity

Physicians, nurses, emergency medical technicians, and the general public participated in the FGDs. The general public in this study are defined as persons with no certificate or license in the healthcare profession. Six to eight study participants attended each group. The healthcare providers who participated in the study were personal contacts of the authors, whereas Gallup Korea selected the general public participants, all of whom were interested in climate and environmental issues. Therefore, in terms of reflexivity, the team assumed that the general public participants in this study would discuss the issues of the study more frankly than the healthcare providers. None of the healthcare providers approached by the authors refused to take part in the FGDs. However, the team could not identify the number and characteristics of the general public who refused to participate in the FGDs because the general public was recruited on a first-come basis. None of the study participants dropped out.

### Data collection

We performed three separate FGDs. Each FGD was conducted in a room with a one-way mirror towards Gallup Korea. Each FGD was audio and video recorded and lasted about two hours on average. The FGDs were led by one professional moderator from Gallup Korea according to semi-structured guidelines. The guidelines were developed to deal with various aspects of the utilization of meteorological information in the healthcare setting. The guidelines are detailed in Table 1.

**Table 1:** Guidelines for conducting focus group discussions

Procedure	Key questions
Ice breaking	• Explanation of the goal and procedures of the research - Self-introduction of the participants
Interest in climate change and health	• Interest in the effects of recent climate change on health • Experience with the effects of recent climate change on health
Disease-specific awareness of effects of climate change	• Representative experience with meteorology-related disease
Conditions of its application and need for meteorological information in the patient care process	• Aspects of meteorological information application in the patient care process • Recognition of the need for meteorological information utilization in the patient care process • Meteorological information and provision method for future needs
Discussion conclusion	• Other questions • Resolution of the meeting

### Analysis

The audio recordings from the FGDs were transcribed verbatim. The verbatim transcriptions and field notes were analyzed based on content analysis [22]. First, one of the authors (MO) thoroughly read the transcriptions and field notes, coded them, and sorted the codes into categories by considering the similarities and relationship among codes. Another author (EYC) reconfirmed and modified the codes and their categories. Disagreements with the results of the analysis were resolved by reiterative analysis. Data saturation was confirmed when the two coders (MO and EYC) identified no additional codes from the transcriptions and field notes. Finally, all the authors reviewed the codes and their categories. The transcripts and results of the analysis were not returned to the study participants for feedback. NVivo 11 software was used for the analysis [23].

### Ethics approval and consent to participate

This study was approved by the institutional review board of UUH (2016–10-011). Prior to enrollment, we explained the objectives and procedures of this study to the participants and obtained informed consent from them. Each participant received one hundred thousand won for joining the FGD.

## Results

A total of 20 individuals—6 physicians, 4 nurses, 3 emergency medical technicians, and 7 members of the general public—participated in the FGDs (Table 2). The specialties of the six physicians were as follows: Emergency medicine, neurosurgery, pediatrics, internal medicine, otolaryngology, and preventive medicine. All the physicians have worked at an acute hospital care setting in the city. Analyses of the transcriptions and field notes derived 115 codes, which were classified into three categories and seven subcategories (Additional file 1). The category and subcategory structures and their main contents are shown in Table 3. More details are provided below.

**Table 2:** Characteristics of the study participants in each group

	First group	Second group	Third group
Occupation or specialty	Physicians 6 - #1: Emergency medicine - #2: Neurosurgery - #3: Pediatrics - #4: Internal medicine - #5: Otolaryngology - #6: Preventive medicine	Outdoor activities: 4 Indoor activities: 3	Emergency room nurse: 2 Emergency medical technicians: 3 Domiciliary nurse: 2
Gender	Man: 4 Woman: 2	Man: 3 Woman: 4	Man: 3 Woman: 4
Age group	30s: 3 40s: 3	30s: 2 40s: 3 50s: 2	30s: 4 40s: 3

**Table 3:** Categories and subcategory structures and their main contents

Category	Subcategory	Main contents
Effects of climate change on health	Direct and indirect effects of climate change on health	• The participants are experiencing an aggravation of their existing disease symptoms, apart from an increased occurrence of climate change-related disease
	Groups vulnerable to climate change	• Patients with underlying diseases, the elderly, and children are vulnerable to climate change • Climate change-related disease incidence and management of prior disease outbreak can differ according to socioeconomic level
The need for recognition of meteorological information utilization	Lack of interest in meteorological information utilization	• Insufficient interest in meteorological information utilization in the actual medical field
	Meteorological information utilization is not the prime patient care issue	• Meteorological information utilization is not the prime issue in the patient care process
	Need to use meteorological information in the patient care process	• Provision of meteorological information to patients in the patient care process will particularly boost rapport with patients • It is necessary to obtain meteorological information in advance to prepare for patients’ questions
Plan for application of meteorological information utilization in the patient care process	The provision method of meteorological information	• Many healthcare providers believed that it would be more useful to offer meteorological information directly to patients, not through healthcare providers, but the general public participants preferred that meteorological information be delivered by healthcare providers such as physicians
	Necessary improvement in the use of meteorological information in the patient care process	• To expand the utilization of meteorological information in the patient care process, meteorological information should be applied to fields and subjects expected to benefit most from the information • Regarding healthcare providers’ participation in meteorological information utilization, evidence of the effects of meteorological information should be demonstrated, and a reformation of the medical system is required to improve the busy medical environment

### Effects of climate change on health

#### Direct and indirect effects of climate change on health

Besides healthcare providers, the general public has directly and indirectly experienced the effects of an increase in various diseases due to climate change. In particular, most of the participants have experienced an aggravation of their existing disease symptoms in addition to an increase in the occurrence of climate change-related diseases. Nonetheless, the recognized health effects of climate change were not based on specific epidemiological data, but rather the healthcare providers’ healthcare experience or personal judgment. Climate change factors affecting health include air temperature, relative humidity, precipitation, and air pressure.

*General public 2: But it could be true if I turn into what you said I think. I know this person and she isn’t that old—this is my sister’s mother-in-law I am talking about—but she collapsed from a hemorrhagic stroke because it was hot during the summer and she passed away afterward. But I heard about this from the news later, like when you exercise, you tend to get this prickly heat during the summer, but back then you more easily got prickly heat when you were sweaty and dried off, but it becomes itchy and reddish, and a more serious type of skin disease if you scratch it accidentally. I believe there certainly are diseases that are affected by the climate change-related weather conditions around us, just like the skin condition and the hemorrhagic stroke I just mentioned.*

#### Groups vulnerable to climate change

Most participants believed that there were individual discrepancies in the believed health effects of climate change. Patients with underlying diseases, such as the elderly and children, were considered groups that are especially vulnerable to climate change. Climate change-related disease incidence and management of prior disease outbreaks can differ according to socioeconomic level as mentioned by some physicians and nurses.

*Physician 3: There already is a correlation between the socioeconomic level and this disease incidence.*

*Moderator: There already is?*

*Physician 3: Yes. I think there is something like that.*

*Physician 4: In fact, people like us working indoors don’t get heatstroke.*

*Physician 3: Yes, that does not really happen.*

*Physician 4: And people with a higher socioeconomic level would not be likely to get infectious diseases. And myocardial infarction and angina pectoris are related in an internal medical sense. I am not so sure about asthma. Asthma is related to personal allergic constitution.*

*Physician 3: I do not know about asthma specifically, but you have a higher chance of getting atopic skin with a lower economic level.*

*Physician 2: They can’t manage it because of their economic status, and they want to take medication even though they don’t have time for it, and it occurs easier because they can’t manage the medication. A person waking up at 9 am and taking his time to get ready and a person who works as a security guard… when they are exposed to extreme weather, there would be a different impact. It’s different for a person who stands a lot as well. In [my] practice, I analyzed the socioeconomic status of osteoporosis patients, and you see more patients of a lower socioeconomic class in the osteoporosis patient group. They get more fractures. Same with osteoporosis.*

*Physician 6: Rich people in the Middle East said something similar, but it’s true here as well. Extremely affluent people can control meteorological phenomena much better. It’s the environment that you are exposed to. For example, if you are hot, you can turn on A/C. Seems like people with a tight budget can’t do that. For example, people who live in a doss-house might sense a slight temperature increase to a greater extent.*

### The need for the recognition of meteorological information utilization

#### Lack of interest in meteorological information utilization

A considerable number of participants felt that there is an insufficient interest in meteorological information utilization in the patient care process. This opinion was more commonly expressed by healthcare providers, particularly physicians, than by the general public participants. Several physician participants believe that the lack of interest in meteorological information utilization is due to the relatively recent emergence of climate change and disease relation research in Korea. Furthermore, the participants mentioned that meteorological information is not employed in real medical fields because the emphasis of meteorological information utilization is only on the surveillance of increases in climate change-related patients.

*Moderator: So, you do not normally use the information when giving care?*

*Physician 4: I have not yet used it.*

*Moderator: I see. Looking for the meteorological information for the patient care process... Physician 5 said he has some experience looking for the meteorological information, is there anybody else with this experience?*

*Physician 3: You mean for patient care?*

*Moderator: Yes. Not just passively watching the weather news and saying “Oh alright,” but actively and proactively researching it first.*

*Physician 4: I guess it is related to the patients, you see. I didn’t specifically look it up in great depth because I only see diabetes and thyroid patients professionally. I assume that physicians who examine chronic diseases or diseases that are less sensitive to temperature or whatever would not specifically look it up.*

### Meteorological information utilization is not the prime patient care issue

Several physician participants felt that meteorological information utilization is not a prime concern during patient care. In addition, the provision of advice employing meteorological information would present some difficulties in busy healthcare environments. Nurses and emergency medical technicians also felt it would be difficult to provide meteorological information due to the healthcare environment. One emergency medical technician recognized that an inordinate provision of meteorological information could pose obstacles in the work environment. A few of the general public participants also suggested a limitation on the utilization of meteorological information during patient care for the same reasons.

*Nurse 4: As you mentioned, if a patient is provided the information before discharge, then trust will be created, and [such advice] is possible in an outpatient clinic, but it’s hard to give advice in the ER because of urgent situations.*

*Emergency medical technician 3: Wouldn’t it be nice to tell it to a discharging patient in the ER? You do tell them some things when you discharge them anyway.*

*Nurse 4: We are short staffed even for informing patients about medication.*

*Emergency medical technician 1: On top of that, just add “it’s supposed to be cold today, so get home safe.”*

*Nurse 2: We can greet them, but they don’t recognize it if we give too much information. And they miss important things.*

*Nurse 4: We have a lot to say and they don’t understand how to take medication, like the explanations they need when they are discharged, so we have to do it more than once. Of course, it would be nice to do it, but it is difficult in reality.*

#### Need to use meteorological information in the patient care process

Most of the general public participants believed that there is a need for meteorological information in the patient care process. They believe that people would appreciate attempts by physicians or healthcare providers to provide meteorological information to prevent disease incidences or relapses. Moreover, the general public participants have been applying various types of research meteorological information to their health management in their daily lives. For example, one general public participant has been seeking ambient air quality information to care for allergic rhinitis, and another has been maintaining the thermostat in her residence for temperature decline. In particular, several participants believe that the provision of meteorological information to patients undergoing care would boost rapport with patients. A considerable number of the general public participants felt that a physician who provides meteorological information is paying careful attention to even minor details, which would increase trust. A number of healthcare providers have been offering meteorological information in the patient care process for the same purpose, and patient experiences verified its ability to help form amicable relationships. Furthermore, some physician participants claimed that it is necessary to obtain meteorological information in advance to prepare for patients’ questions.

*Emergency medical technician 2: Wouldn’t a patient or caregiver gain some trust if you say one or two words like that because they feel warmth? If you say something like “It’s cold today, so don’t go out,” then as a listener, he or she could feel “This person must care for me.” Then, wouldn’t the trust increase?*

### Plan for application of meteorological information utilization in the patient care process

#### The provision method of meteorological information

The healthcare providers and general public participants expressed differences in how the actual meteorological information should be provided. Many healthcare providers believe that it would be more useful to offer meteorological information directly to patients, not through healthcare providers. However, the general public participants prefer that meteorological information be delivered by healthcare providers, such as physicians. The reasoning was that the advice given by a healthcare provider to a patient would be perceived more positively than information provided by family members. In addition, social media or mobile phone text messaging was the favored form of meteorological information delivery.

*General public 2: Not only that, but all types of medical information can be shared* via *Kakaotalk (an instant messenger application for mobile phones) or some other messenger. I think you get something out of it. You share so much. “You gotta do this for that”... “If your teeth ache, do this”... “For this, go to a hospital.”*

*(break)*

*Moderator: What would be the most convenient method to receive a message such as “the weather has worsened, so watch out like this?”*

*Everyone: Kakaotalk.*

*General public 4: Kakaotalk or text.*

#### Necessary improvements in the use of meteorological information in the patient care process

To expand the utilization of meteorological information in the patient care process, the participants suggested that meteorological information be applied to fields and subjects expected to get the most benefit out of the information. In other words, they felt that the meteorological information should be provided to subjects vulnerable to climate change in terms of prevention. Moreover, from the participants’ perspective, the use of meteorological information could be misappropriated for perfunctory service, whereas it could help to form a rapport with patients in the patient care process. Furthermore, in contemplation of the healthcare providers’ participation in meteorological information utilization, evidence of the effects of meteorological information should be demonstrated, and a reformation of the medical system to improve the busy medical environment was proposed by some of the participants.

*Physician 1: There should be results showing the disease worsening, demonstrating correlation, but there is no research paper on outcome improvements because of the information offered that gives that specific information.*

*Physician 3: In fact, with a verified reference, I can tell a person who has to go outdoors to work to not go because it’s cold. But there’s not enough (evidence) for that.*

## Discussion

We performed three FGDs with six physicians, four nurses, four emergency medical technicians, and seven members of the general public to examine their perceptions regarding the utilization of meteorological information in healthcare. The key findings from this study were classified into three categories as shown in Table 3. Similar to previous studies [24, 25], most of the participants, including the healthcare providers, have noticed an increase in the occurrence of climate change-related disease and an aggravation of disease symptoms. In particular, most participants were concerned that health disparities will be worsened because the incidence of climate change-related diseases and their management can differ according to socioeconomic status. Climate change can aggravate health disparities [8] and it is therefore important to quickly identify the groups vulnerable to climate change and manage vulnerabilities to the health effects of climate change [26]. One of the strategies to manage vulnerabilities would be to reduce social isolation because vulnerable groups, such as patients with underlying disease and the elderly, often lack support from social networks to help them manage and recover from climate change [27].

Although most healthcare provider participants acknowledged that the health effects of climate change exist, several healthcare provider participants, especially physicians, expressed insufficient interest in meteorological information utilization in the patient care process. Several physician participants also mentioned that meteorological information utilization is not a prime concern during patient care. They felt this way because it is not easy for healthcare providers to give advice employing meteorological information to patients in a busy healthcare environment. These findings were similar to those of Anåker et al., which identified an incongruity between climate and environmental issues and the daily work of nurses [20]. Thus, climate and environmental issues were not considered a primary problem in lifesaving and hectic healthcare situations.

In contrast, most of the general public participants agreed on the need for the utilization of meteorological information in the patient care process. Indeed, they have already sought and used meteorological information for their own health management. Therefore, the general public might ask healthcare providers for meteorological information, such as the relationship between disease occurrence or exacerbation and meteorological information and the manner of climate-sensitive disease control. Furthermore, this qualitative study showed that the provision of meteorological information to patients during care will boost rapport between healthcare providers and patients. These results will require fundamental changes in the healthcare providers’ attitudes and behaviors regarding the utilization of meteorological information in healthcare. Healthcare providers need to improve their ability to address the health-related impact of climate change and play an active role in alerting and educating the general public about the health risks of climate change [24, 28].

In order to boost the utilization of meteorological information in the patient care process, paradoxically, empirical evidence on the effects of the provision of meteorological information should be generated. However, empirical evidence on the effects of health forecasting is scarce and equivocal; although, health forecasting is ideal for healthcare services. In the case of *Healthy Outlook*, Sarran et al. [13] reported that participation in the *Healthy Outlook* service significantly decreased the hospital admission rate, but Bakerly et al. [29] determined that *Healthy Outlook* had no effect on reducing hospital admissions. In addition, Steventon et al. [14] reported that *Healthy Outlook*, although failing to decrease admission rates, did decrease the mortality rate. However, these studies were conducted in the United Kingdom and focused on COPD patients, and it is hard to find studies evaluating the effects of health forecasting in other countries, including Korea. As mentioned by the participants in this study, the aim should be to prioritize the provision of meteorological information to subjects according to their vulnerability to climate change.

If a new health forecasting service is developed, it will be important to give practical information to the general public, including patients, so they feel that the meteorological information provided is relevant. The common causes of dissatisfaction with health forecasting services are that the information lacks detail and value and can be easily obtained from other standard sources [15]. Furthermore, the provision of meteorological information should not be regarded as a perfunctory service. Therefore, effective communication of tailored meteorological information from healthcare providers to the general public will be a cornerstone of effective and sustainable health forecasting services [12].

The main limitation of this qualitative study is that the findings reflect certain points of view in terms of geographic area, age group, and the physicians’ specialty and practice setting. In particular, although climate change is a global issue, the extent of the problem and the views on its significance may differ. Similar studies should be performed in other countries to confirm the results of this qualitative study. In addition, we cannot rule out the possibility that healthcare provider participants were not completely open regarding the issue in terms of reflexivity because the healthcare provider participants were specifically recruited via personal contacts of the authors. However, we thought the potential for reflexivity was limited because the opinions of the healthcare provider participants on the utilization of meteorological information in the healthcare setting were less positive than those of the general public participants.

## Conclusions

In conclusion, both healthcare providers and the general public acknowledge that the health effects of climate change have become a reality. Although several healthcare provider participants express insufficient interest in meteorological information utilization in the patient care process, most of the general public participants agreed that meteorological information should be used in the patient care process. The provision of meteorological information to patients in the patient care process is expected to benefit not only the management of climate-sensitive diseases, but will also build rapport between healthcare providers and patients. Meteorological information should be provided to groups vulnerable to climate change and its effects should be evaluated in terms of effectiveness and inequality. The main findings from this study will be helpful in countries and institutions trying to introduce health forecasting services.

## Additional file

Additional file 1: Table S1. Categories, Subcategories and Main Contents. (DOCX 16 kb)

## Abbreviations

COPD: Chronic obstructive pulmonary disease
FGD: Focus group discussion
