# Correction to: Perceptions regarding utilization of meteorological information in healthcare in Korea: a qualitative study

**DOI:** 10.1186/s40557-018-0234-z

**Published:** 2018-05-03

**Authors:** Minsu Ock, Eun Young Choi, Inbo Oh, Seok Hyeon Yun, Yoo-Keun Kim, Hyunsu Kim, Min-Woo Jo, Jiho Lee

**Affiliations:** 1 Department of Preventive Medicine, Ulsan University Hospital, University of Ulsan College of Medicine, Ulsan, Republic of Korea; 2 Department of Preventive Medicine, University of Ulsan College of Medicine, Seoul, Republic of Korea; 3 Environmental Health Center, University of Ulsan College of Medicine, Ulsan, Republic of Korea; 4 Department of Occupational and Environmental Medicine, Ulsan University Hospital, University of Ulsan College of Medicine, 877 Bangeojinsunhwan-doro, Dong-gu, Ulsan, 44033 Republic of Korea; 5 Department of Atmospheric Sciences, Pusan National University, Busan, Republic of Korea

**Correction**

After publication of the original article [1] the authors flagged that the Funding information in the Declarations was incorrect.

The original manuscript reads:

**Funding:**

This study was funded by the Korea Ministry of Environment (MOE) as “the Environmental Health Action Program” (grant number: KMIPA2015–8016).

However, the following statement should have been included at the time of publication:

**Funding:**

This work was funded by the Korea Meteorological Administration Research and Development Program under Grant KMIPA2015-8062.
