# Supplementary material for: Perceptions regarding utilization of meteorological information in healthcare in Korea: a qualitative study
Source: Ann Occup Environ Med. 2018 Feb 1;30:8. doi: 10.1186/s40557-018-0214-3 (PMC5793381; doi:10.1186/s40557-018-0214-3)
Supplement: Additional file 1: Table S1. — Categories, Subcategories and Main Contents. (DOCX 16 kb) [file 40557_2018_214_MOESM1_ESM.docx]

부록 표 1. 범주, 하위범주 및 주요 내용

| 범주 | 하위범주 | 주요 내용 |
| --- | --- | --- |
| 기후 변화에 따른 건강 영향 | 기후 변화로 인한 건강 영향을 직간접적으로 느낌 | • 기후 변화로 관련 질병의 발생이 증가하는 것뿐만 아니라 기존의 질병의 증상이 악화됨을 경험하고 있음. |
|  | 기후 변화에 취약한 집단 | • 기저 질환이 있는 환자들, 노인들, 아이들이 기후 변화에 취약함.  • 사회경제적 수준에 따라 기후 변화로 인한 질병 발생에 차이가 있을 것이고, 이미 발생한 질병의 경우에도 사회경제적 수준에 따라 기후 변화에 대처하는 정도가 다를 수 있음. |
| 기상  정보 활용의 필요성 인식 | 기상 정보 활용에 아직 관심이 부족함 | • 기상 정보를 실제 진료 현장에 활용하는 것에 대한 관심이 부족함. |
|  | 기상 정보 활용이 주된 진료 문제는 아님 | • 진료 과정에서 기상 정보를 활용하는 것이 주된 진료 문제는 아님. |
|  | 진료 과정에서 기상 정보 활용이 필요함 | • 진료 과정에서 기상 정보를 환자에게 제공하는 것은 환자와의 좋은 관계를 형성시키는 데에 도움이 될 것임.  • 기상과 질병 간 연관성에 대한 환자의 질문에 대비하여 관련 지식을 알고 있는 것이 필요함. |
| 진료 과정에서 기상 정보 활용 방안 | 기상 정보 제공 방식 | • 의료진 참여자들은 의료인을 거치지 않고 환자에게 직접 기상 정보를 제공하는 것이 더 유용할 것이라고 생각하였지만, 일반인 참여자들은 의사 등의 의료진이 기상 정보를 제공하는 것을 선호하였음. |
|  | 기상 정보를 진료 과정에서 활용하기 위하여 필요한 개선 사항 | • 기상 정보 활용이 유용할 것으로 예상되는 영역 및 대상에서부터 기상 정보의 활용을 시도해보는 것이 좋겠음.  • 진료 과정에서의 기상 정보 활용에 대한 의료진의 참여를 유도하기 위해서는 우선 기상 정보 제공의 효과에 대한 근거부터 마련해야 하고, 바쁜 진료 환경을 개선시킬 수 있도록 의료 제도의 개선이 선행될 필요가 있음. |
